# Differential Effects of a Nap on Motor Sequence Learning-Related Functional Connectivity Between Young and Older Adults

**DOI:** 10.3389/fnagi.2021.747358

**Published:** 2021-10-28

**Authors:** Zhuo Fang, Dylan M. Smith, Genevieve Albouy, Bradley R. King, Catherine Vien, Habib Benali, Julie Carrier, Julien Doyon, Stuart Fogel

**Affiliations:** ^1^School of Psychology, University of Ottawa, Ottawa, ON, Canada; ^2^Department of Movement Sciences, KU Leuven, Leuven, Belgium; ^3^Department of Health and Kinesiology, College of Health, University of Utah, Salt Lake City, UT, United States; ^4^Department of Psychology, University of Montreal, Montreal, QC, Canada; ^5^Functional Neuroimaging Laboratory, INSERM, Paris, France; ^6^Centre for Advanced Research in Sleep Medicine, Hôpital du Sacré-Coeur de Montreal, Montreal, QC, Canada; ^7^McConnell Brain Imaging Centre, Montreal Neurological Institute, McGill University, Montreal, QC, Canada; ^8^Department of Neurology and Neurosurgery, Montreal Neurological Institute, McGill University, Montreal, QC, Canada; ^9^Functional Neuroimaging Unit, Centre de Recherche Institut Universitaire de Gériatrie de Montréal, Montreal, QC, Canada; ^10^Department of Psychology, University of Montreal, Montreal, QC, Canada; ^11^Sleep Unit, University of Ottawa Institute of Mental Health Research at The Royal, Ottawa, ON, Canada; ^12^University of Ottawa Brain and Mind Research Institute, University of Ottawa, Ottawa, ON, Canada

**Keywords:** sleep, functional connectivity, motor sequence learning, MRI, resting state

## Abstract

In older adults, motor sequence learning (MSL) is largely intact. However, consolidation of newly learned motor sequences is impaired compared to younger adults, and there is evidence that brain areas supporting enhanced consolidation *via* sleep degrade with age. It is known that brain activity in hippocampal–cortical–striatal areas is important for sleep-dependent, off-line consolidation of motor-sequences. Yet, the intricacies of how both age and sleep alter communication within this network of brain areas, which facilitate consolidation, are not known. In this study, 37 young (age 20–35) and 49 older individuals (age 55–75) underwent resting state functional magnetic resonance imaging (fMRI) before and after training on a MSL task as well as after either a nap or a period of awake rest. Young participants who napped showed strengthening of functional connectivity (FC) between motor, striatal, and hippocampal areas, compared to older subjects regardless of sleep condition. Follow-up analyses revealed this effect was driven by younger participants who showed an increase in FC between striatum and motor cortices, as well as older participants who showed decreased FC between the hippocampus, striatum, and precuneus. Therefore, different effects of sleep were observed in younger vs. older participants, where young participants primarily showed increased communication in the striatal-motor areas, while older participants showed decreases in key nodes of the default mode network and striatum. Performance gains correlated with FC changes in young adults, and this association was much greater in participants who napped compared to those who stayed awake. Performance gains also correlated with FC changes in older adults, but only in those who napped. This study reveals that, while there is no evidence of time-dependent forgetting/deterioration of performance, older adults exhibit a completely different pattern of FC changes during consolidation compared to younger adults, and lose the benefit that sleep affords to memory consolidation.

## Introduction

As we age, the brain activity underlying our sleep changes drastically. In youth, sleep-dependent physiological brain processes support important cognitive functions such as skill learning and memory, however, the ability for sleep to enhance learning and memory begins to deteriorate in middle age and beyond (Mander et al., 2013; Scullin and Bliwise, 2015). Models of sleep-enhanced learning consolidation propose that during sleep, the reduced need to process incoming stimuli creates ideal conditions for reactivation of hippocampus-dependent memory traces, and the subsequent transfer of newly acquired information to subcortico-cortical networks (Boutin and Doyon, 2020). During sleep, events such as sleep spindles support the process of hippocampus-dependent consolidation of newly acquired information. According to this framework, age-related changes in sleep might not provide the same benefit to the normal consolidation of hippocampal memory traces. However, the specifics of how aging causes the process of sleep-enhanced memory consolidation to change are still unclear.

Studies comparing healthy young and older adults have consistently shown differences in both the macro-and micro-architecture of sleep. Compared to younger participants, older adults spend more time transitioning from wake to sleep, and spend less time in non-rapid eye-movement (NREM) sleep stage 2 (N2), slow-wave sleep (SWS), and rapid eye movement (REM) sleep; stages wherein memory consolidation occurs (Diekelmann and Born, 2010). In addition, the electrophysiological features of sleep such as sleep spindles are also reduced with age. Spindles, which are hallmarks of N2 sleep and are thought to support memory consolidation, are reduced in terms of their number, amplitude, duration, and tend to occur at slower frequencies (Knoblauch et al., 2005; Fogel et al., 2012; Martin et al., 2013) in older individuals compared to young adults. Similarly, other neural oscillation events, such as K-complexes and slow waves, are also reduced with age (Crowley et al., 2002; Carrier et al., 2011). Thus it is possible that the observed changes in macro-and microstructural sleep partially explain the phenomenon of degraded sleep-enhanced memory consolidation with age.

Sleep is known to be important for supporting the consolidation of procedural memory, particularly motor sequence learning (MSL), which corresponds to the ability to precisely and reliably execute a sequence of learned finger movements. While many studies have identified a consolidating effect of sleep on MSL (see King et al., 2017a for review), meta-analyses have identified publication bias in the literature (Pan and Rickard, 2015), with some studies specifying a stabilizing rather than enhancing effect of sleep on MSL (Nettersheim et al., 2015). Overall, when compared to wake, sleep is associated with a small positive effect for motor memory consolidation, and a short daytime nap has nearly the same consolidating effect as does a full night of sleep (Schmid et al., 2020). Neuroimaging studies comparing sleep to wake in motor memory consolidation have found evidence that striatal activity during sleep may be particularly important for moderating sleep’s effect on MSL. For example, both spindle amplitude and cortico-striatal activation have shown to be positively associated with motor sequence memory consolidation in young adults (Barakat et al., 2013). The magnitude of offline gains in performance (reflecting consolidation) in young adults is also related to the extent of the enhancement in functional connectivity (FC) in the putamen, as this same brain region is reactivated during sleep spindles during post-learning sleep (Fogel et al., 2017; Vahdat et al., 2017). Furthermore, analysis of post-MSL resting state FC in young adults has shown that cortico-striatal communication patterns associated with MSL are reorganized and strengthened during a period of NREM sleep, but not wake, emerging as stronger cortico-striatal connectivity compared to the initial memory-trace (Vahdat et al., 2017). It is not known if this is also the case for older adults.

Importantly, young and older adults show similar rates of learning in MSL, suggesting that motor skill learning is intact in older populations (Fogel et al., 2014). However, older adults show a specific deficit in the consolidation of newly acquired motor sequences after a period of sleep (Spencer et al., 2007; Wilson et al., 2012). This age-related reduced benefit of sleep for memory consolidation has been associated with a reduction in sleep spindles, as well as reduced change in striatal activity across the retention period (Fogel et al., 2014).

In older adults, greater cortico-striatal activation during initial learning of an MSL task has been associated with better consolidation, but only when participants are given the chance to nap between training and retest (King et al., 2017b). Moreover, older adults show decreases in MSL task-relevant areas from learning to retest, whereas young adults show increased activity in these areas (Fogel et al., 2014). It is possible that communication between hippocampal, striatal, and cortical brain areas during sleep may degrade with age, and may explain the lost benefit of sleep for memory consolidation. In older adults, the mechanism of sleep-enhanced consolidation may fail to efficiently transfer newly learned information to task-relevant brain areas, interrupting the process of memory reactivation, and limiting consolidation.

It has been proposed that sleep-enhanced memory consolidation proceeds *via* spindle-induced inter-regional reactivation of the same cortical and subcortical areas that were functionally active during initial learning (Boutin and Doyon, 2020). However, it is not known whether the process of consolidation is associated with detectable changes in resting state FC, and furthermore, whether these potential changes are lost with age. Thus, the present study investigated the effects of a nap vs. a period of wake on motor memory consolidation in young and older adults. Using functional magnetic resonance imaging (fMRI), we assessed how resting state functional communication changes after motor learning and after a retention interval consisting of either sleep or wake. If consolidation of a newly acquired motor sequence is enhanced by sleep-dependent changes in functional brain connectivity, then differences in resting state functional communication should be observed after a nap compared to a similar period of wake over the retention period. We further hypothesized that these changes would be unique to younger adults and not necessarily observed in older adults, due to age-related changes in FC between brain regions involved in motor memory consolidation. Finally, we hypothesized that any relationship between FC changes and MSL performance gains would be stronger, or limited to young participants, as the degradation of sleep-dependent consolidation mechanisms should prevent consolidation-supporting FC changes from being observed in older adults.

## Materials and Methods

### Participants

Participant data from the current study comes from a previously unpublished subset of resting sate fMRI data from Fogel et al. (2014). Groups of young healthy volunteers (*n* = 37, 23 females) between the age of 20 and 35 years (24.0 ± 3.8) and older healthy individuals (*n* = 49, 32 females) between the age of 55 and 75 years (62.6 ± 5.0) were recruited for the study. Ethical and scientific approval was obtained from the Ethics Committee at the “*Institut Universitaire de Geriatrie de Montreal*,” Montreal, QC, Canada, and informed written consent was obtained prior to entering the study. All participants met the inclusion and exclusion criteria following the study protocol. Initial screening was completed through a telephone pre-screening interview and battery of online questionnaires for each participant. From these questionnaires, participants were included if they were right-handed (scored >40 on the Edinburgh Handedness Inventory; Oldfield, 1971), non-smokers, non-heavy drinkers (>14 alcoholic drinks per week), prescription medication-free, reported they had a normal body mass index (≤25) and had never been diagnosed with any neurological, psychological, psychiatric, or sleep disorders. Participants also had to score ≤8 on the short version of the Beck Depression Scale (Beck et al., 1974) and ≤8 on the Beck Anxiety Scale (Beck et al., 1988) to be included. In addition, older participants were only included if they scored ≥24 and ≥25 on the Mini Mental State Examination and Montreal Cognitive Assessment Battery, respectively (Cockrell and Folstein, 1988; Tombaugh and McIntyre, 1992). Participants were eligible for the study only if they had no previous formal training as a typist or musician, nor considered themselves to be expert video game players. Additionally, participants who worked at night or had taken a trans-meridian trip ≤3 months prior to the study were not allowed to participate. In order to avoid recruiting participants that would be unable to nap during the day, “non-nappers”; those who never nap, intentionally avoid napping, and do so because of excessive sleep inertia afterward were excluded. Finally, participants had to score between 30 (i.e., “moderate evening”) and 70 (i.e., “moderate morning”) on the Horne Ostberg Morningness-Eveningness Scale (Horne and Ostberg, 1976) to exclude either “extreme morning” or “extreme evening” types, in order to avoid the potentially confounding effects of differences in circadian-related factors and sleep pressure between individuals.

In addition to the pre-screening criteria, all participants were required to limit alcohol and caffeine consumption (≤1 serving/day) and abstain from nicotine during the experiment to be included. They were also required to keep a regular sleep–wake cycle (bed-time: 22:00–1:00, wake-time: 07:00–10:00) and to abstain from daytime naps to be included. Participants’ sleep–wake cycles were monitored throughout the experiment with the use of a sleep journal and an actigraph to ensure compliance with sleep–wake instructions. Participants who did not comply with these instructions were excluded from further participation in the study.

**FIGURE 1:**
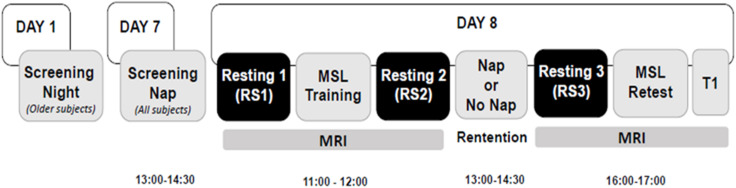
Experimental design. Older participants underwent an overnight screening night (Day 1), 1 week prior to the screening nap. Then all participants underwent a screening nap (Day 7) from 13:00 to 14:30 and returned on the following day for the remainder of the experimental protocol (Day 8), on which day two MRI scan sessions were performed. The morning MRI session took place at 11:00 and lasted ∼45 min. It consisted of an MSL task training session, and two resting scans, one was before the MSL session (RS1), the other was immediately after the MSL session (RS2). Participants were then assigned to either the Nap or No Nap condition. The retention interval began at 13:00 and lasted 90 min, with an additional >1 h for recovery from any sleep inertia effects. The afternoon MRI scanning session began at 16:00 and lasted ∼45 min, which comprised a resting scan (RS3) session followed by the MSL task retest session and a T1-weighted anatomical scan (T1).

Eligible participants who passed the initial screening underwent a 90-min acclimatization nap beginning at 13:00, which was scheduled prior to the beginning of the study (see Figure 1, “Day 7”). In addition, older participants underwent an overnight polysomnographic (PSG) screening night (see Figure 1, “Day 1”) to exclude participants with an apnea–hypopnea index (AHI) >5 and a periodic limb movement (PLM) index >10. In total, 19 older participants were excluded before completing the protocol; 10 were excluded for an elevated AHI or PLM index, 2 for insufficient sleep during the acclimatization nap, 2 for MRI safety concerns and 5 voluntarily withdrew from the study. A total of seven young participants were also excluded before completing the protocol; six voluntarily withdrew from the study and one unexpectedly had a vascular abnormality revealed upon visual inspection of the T1-weighted MRI scan and assessed by a certified neuro-radiologist.

From those who met the inclusion/exclusion criteria and completed the protocol, groups of young (*n* = 30, 17 females) and older adults (*n* = 30, 21 females) were included in the study and randomly assigned to either the Nap condition [*n* = 15 Young Nap (YN); *n* = 15 Older Nap (ON)] or No Nap condition (*n* = 15 Young No Nap (YNN); *n* = 15 Older No Nap (ONN)]. Of these 60 participants, two from the YN condition were excluded from the data analyses. One participant reportedly suffered from severe sleepiness in the last five blocks of training and succumbed to extreme tiredness during practice, thus producing lapses in performance during the training session. A second subject did not comply with the instructions given throughout the experiment.

### Experimental Design

The overall experimental design is shown in Figure 1. The MRI test session took place on Day 8 following the screening night (Day 1) and the acclimatization nap (Day 7). All participants were asked to arrive in the laboratory at 10:30 and the MRI scan started at 11:00. During the morning session of the MRI scan (11:00–12:00), two resting state scan sessions were performed before (RS1) and immediately after (RS2) the MSL training session. Participants were then randomly assigned to either the Nap or No Nap condition for the 90-min retention interval that began at 13:00. A daytime nap protocol was selected to avoid time-of-day effects on performance between young and older participants. Participants in the No Nap condition were required to remain awake during the same 90-min interval and were allowed to read quietly while supine in bed under dim ambient light. PSG measures were recorded in the Nap condition to verify the Nap sleep duration as well as in the No Nap condition to verify that all participants remained awake. If signs of drowsiness (e.g., elevated alpha activity, lower muscle tension, and slow rolling eye movements) were observed in the No Nap condition, the experimenter entered the room to alert the participant. The detailed PSG recording and analysis have been previously reported in these participants (Fogel et al., 2014). The second MRI session took place around 16:00, >1.5 h after the end of the nap or wake period to avoid sleep inertia effects. During this session, the third resting state scan (RS3) was performed before the MSL retest session (see “*Brain Imaging Data Acquisition and Analysis*” for details).

### Finger Sequence Task

The detailed MSL procedure is reported in Fogel et al. (2014) and is thus presented only briefly here. MSL was tested using an adapted version of the five-item sequential (“4-1-3-2-4”) finger-tapping task (Karni et al., 1995), where “1” corresponds to the index finger and “4” to the little finger of the non-dominant (left) hand. An MR-compatible response box (model HH-1 × 4-L; Current Designs, Inc., Philadelphia, PA, United States) comprising four push buttons located in a horizontal row at equal distance from each other was used. Each session consisted of 14 blocks of practice, and each block terminated after participants had produced 60 key presses. Finally, speed was measured by calculating inter-key-press intervals for correct responses. All participants in the analysis performed the sequence with an accuracy above 83.3% overall, corresponding to 10/12 correct sequences per block. Offline gains scores were calculated by taking the percent change from the mean of the last four blocks of the training session, to the mean of the first four blocks of the retest session. A training block (blocks 1–14), by sleep/wake condition (Nap, No Nap), by age group (Young, Older) ANOVA was used to verify that participants were able to improve on the MSL task as a function of practice during the training session. Then, to investigate the impact of sleep on subsequent performance improvements, a sleep condition (Nap, No Nap) by age group (Young, Older) ANOVA was conducted using the gains scores. MRI data acquired while subjects were performing the MSL task was not used for the current analysis, but was analyzed and reported in Fogel et al. (2014).

### Imaging Data Acquisition and Analysis

#### MR Sequence Parameters

Brain images were acquired using a 3.0T TIM TRIO magnetic resonance imaging system (Siemens, Erlangen, Germany) and a 12-channel head coil. For all participants, multislice T2^∗^-weighted fMRI images were acquired during the resting-state sessions, as well as the MSL training and retest sessions, with a gradient echo-planar sequence using axial slice orientation (TR = 2650 ms, TE = 30 ms, FA = 90°, 43 transverse slices, 3 mm slice thickness, 10% inter-slice gap, FoV = 220 mm × 220 mm, matrix size = 64 × 64 × 43, and voxel size = 3.44 mm × 3.44 mm × 3 mm). Each resting-state session lasted ∼6 min (150 volumes). Participants were required to keep eyes-closed during the resting scans. A structural T1-weighted MRI image was also acquired using a 3D MPRAGE sequence (TR = 2300 ms, TE = 2.98 ms, TI = 900 ms, FA = 9°, 176 slices, FoV = 256 mm × 256 mm, matrix size = 256 × 256 × 176, and voxel size = 1 mm × 1 mm × 1 mm).

#### MRI Preprocessing

Preprocessing and FC analysis of resting-state functional images were performed using CONN toolbox v18^1^ (RRID:SCR_009550). Preprocessing using the default pipeline in CONN toolbox was performed, including realignment, co-registration (individual structural images were co-registered to the mean functional image after realignment, segmentation, and normalization (the transformed structural images were then segmented into gray matter, white matter, and cerebrospinal fluid), and smoothing (6 mm FWHM kernel). To remove nuisance signals, the default denoising pipeline in CONN toolbox was applied. Specifically, the signals from white matter and cerebrospinal fluid were regressed out by estimating five potential noise components (Chai et al., 2012). The Friston 24-parameter model was utilized to regress out head motion effects from the realigned data. Scrubbing was performed using artifact detection tools with the threshold for global-signal above 5 (*z*-value) and for subject-motion above 0.9 mm. In addition, linear and quadratic trends were also included as regressors since the BOLD signal exhibits low-frequency drifts. Temporal filtering (0.008–0.09 Hz) was then performed on the time series. To control for the effects of head motion differences across groups, the head motion and scrubbing parameters were included in the first-level as covariates of no interests.

#### Functional Connectivity Analyses

A region of interest (ROI) approach was used to evaluate FC between 15 *a priori* selected ROIs (Supplementary Table 1), which play a crucial role in MSL and sleep-related memory consolidation based on our hypotheses and previous literature. This set of ROIs included multiple regions within cortical, striatal, and hippocampal areas (Doyon and Benali, 2005; King et al., 2017b). The ROIs were defined from the CONN toolbox’s default anatomical atlas (i.e., Harvard–Oxford atlas) and the default networks atlas (Whitfield-Gabrieli and Nieto-Castanon, 2012). To compute FC, the averaged time-series for each ROI from the preprocessed images was extracted. Then, a Pearson’s correlation coefficient for each pair ROIs was calculated and converted to *z* scores using Fisher’s *r*-to-*z* transformation.

Additionally, less constrained, yet more exploratory whole brain seed-to-voxel based FC analyses were performed to confirm the specificity of the hypothesis-driven ROI-to-ROI results. The pre-defined ROIs were used as seeds to calculate the correlation coefficients between the time series of these seed regions and all other voxels in the whole brain. Finally, any significant FC results were followed up to examine if any relationship existed between the strength of the FC between ROIs and offline gains in MSL performance.

#### Group-Level Analyses

Using the ROI-to-ROI approach, our primary aim was to examine: (1) aging differences in learning-related FC changes immediately after MSL training (RS2–RS1), and (2) the interaction effects of age group (Young/Old) × sleep (Nap/No Nap) on post-learning MSL-related FC changes (RS3–RS2). The statistical threshold used was two-sided p*FDR* < 0.05 for ROI-to-ROI results. The same age group × sleep (Nap/No Nap) interaction effects on FC changes (RS3–RS2) were also investigated using seed-to-voxel approach, and the statistical threshold used was uncorrected *p* < 0.005 at the whole brain level, and then false discovery rate (FDR) corrected *p* < 0.05 at the cluster level.

### Associations Between Post-learning Motor Sequence Learning-Related Functional Connectivity Changes (RS3–RS2) and Performance Gains

The ROI-to-ROI approach was applied to explore the relationships between the post-MSL FC changes (RS3–RS2) and performance gains, as well as the nap effects on the relationships in young and older groups, respectively. The individual performance gains were included in the model as covariates of interests. The statistical threshold used was one-sided pFDR < 0.05.

## Results

The detailed sleep architecture data is reported in a previous publication (Fogel et al., 2014). During the 90-min daytime nap, the total sleep time (mean ± SEM) of the young group was 72.08 ± 3.56 (min), and the old group was 46.00 ± 7.26 (min).

### Behavioral Performance

#### Training Session

**FIGURE 2:**
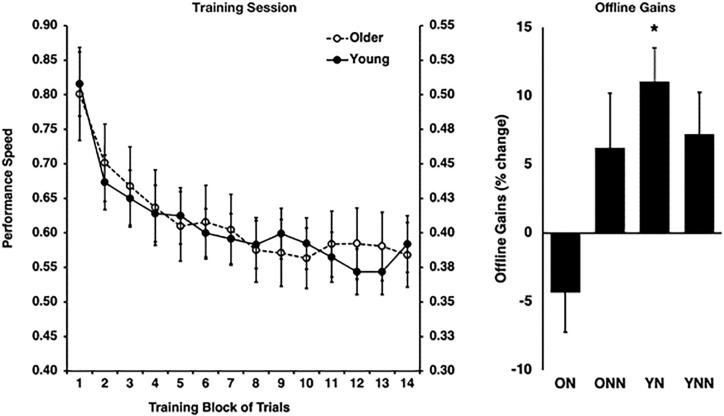
*Left panel*: behavioral performance speed on the motor sequence learning (MSL) task at training for young (right *y*-axis) and older (left *y*-axis) groups. *Right panel*: offline gains in performance (% change last four blocks of training to first four blocks of retest). *Indicates statistically significant (*p* < 0.05) offline gain in the Young Nap condition (YN). YN, Young Nap; YNN, Young No Nap; ON, Older Nap; ONN, Older No Nap.

As expected, this analysis revealed that older adults were slower overall [*F*(1,53) = 14.69, *p* < 0.0001], but that both young [*F*(13,338) = 14.41, *p* < 0.0001] and older adults [*F*(13,251) = 19.93, *p* < 0.0001] improved their performance significantly over the course of the training trials [*F*(13,689) = 32.13, *p* < 0.0001]. Indeed, performance during the training session increased at a rate that significantly followed an inverse function curve in both young [best-fit: *R*^2^ = 0.95, *F*(1,12) = 229.57, *p* < 0.001] and older subjects [best fit: *R*^2^ = 0.96, *F*(1,12) = 259.76, *p* < 0.001]. Furthermore, a Fisher’s *z*-to-*r* transformation revealed that the rate of improvement did not differ between young and older adults (*z* = −0.41, *p* = 0.682), suggesting similar rates of improvement with practice Figure 2). These results suggest that despite slower motor execution in older adults, learning during the training session was comparable between age groups.

#### Training vs. Retest

Then, to investigate the impact of sleep on subsequent performance improvements, a sleep condition (Nap, No Nap) by age group (Young, Older) ANOVA was conducted using the gains scores, which revealed a significant sleep by age interaction effect [*F*(1,53) = 4.87, *p* = 0.032]. Follow-up Holm–Bonferroni *t*-tests revealed that young [*t*(12) = 4.32, *p* = 0.004], but not older [*t*(14) = −1.47, *p* = 0.163], adults who slept showed significant sleep-dependent offline gains in MSL performance (Figure 2). By contrast, there were no significant offline gains in performance in young or older adults in the wake condition (see Fogel et al., 2014 for detailed information).

### Region of Interest-to-Region of Interest Functional Connectivity Results

#### Age Differences in the Functional Connectivity Changes After Motor Sequence Learning (RS2–RS1)

**FIGURE 3:**
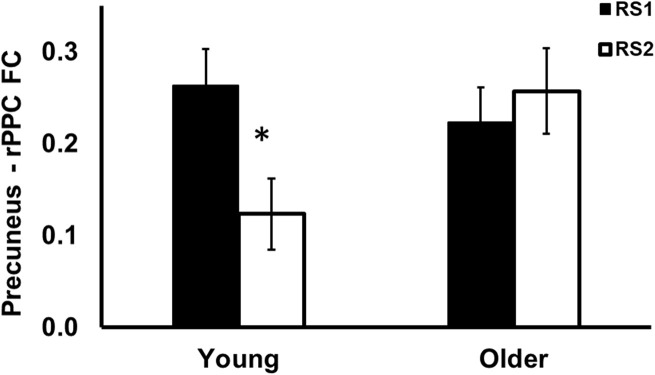
Region of interest analysis of FC between precuneus and right posterior parietal cortex during RS1 and RS2. Significant age × session interaction effect on the FC between precuneus and right posterior parietal cortex. Specifically, the young group showed significant decreased FC between precuneus and right posterior parietal cortex, while no such differences were observed in the older group. *indicates pFDR < 0.05.

As shown in Figure 3, among the 15 ROIs, there was a significant interaction effect of age (Young/Old) × resting state session (RS1/RS2) on FC between the precuneus and the right posterior parietal cortex (PPC) [*F*(1,56) = 9.25, *p* = 0.004]. Specifically, the young group showed decreased FC from baseline to post-training (RS2–RS1) between the precuneus and rPPC [*t*(27) = −3.72, *p* = 0.010], while no such differences were observed in the older group [*t*(29) = 0.8, *p* = 0.93]. No age difference in FC was found during RS1 or RS2.

### Age Group × Sleep Interaction Effects on the Post-learning Motor Sequence Learning-Related Functional Connectivity Changes (RS3–RS2)

**FIGURE 4:**
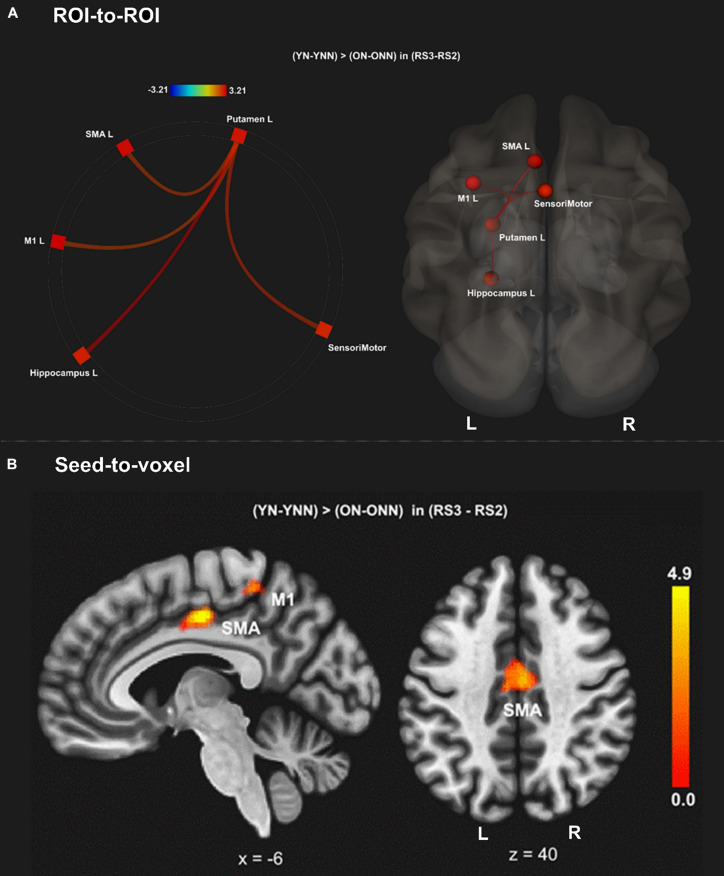
**(A)** Region of interest-to-region of interest results of age × sleep interaction effects on post-MSL FC changes in the cortico-striatal–hippocampal regions from RS2 to RS3. **(B)** Seed-to-voxel results of age × sleep interaction effects on post-MSL FC changes between putamen and motor cortex from RS2 to RS3. The differences between YN and YNN in the FC changes were greater than that between ON and ONN. YN, Young Nap; YNN, Young No Nap; ON, Older Nap; ONN, Older No Nap.

To test our main hypothesis, the impact of age on the benefit of a daytime nap for the consolidation of MSL was investigated using an ROI-to-ROI approach. First, we explored the interaction between age (Young, Older) and sleep condition (Nap, Wake) [(YN–YNN)–(ON–ONN)] in resting state FC changes from immediately following MSL training (RS2) to after the sleep/wake consolidation interval (RS3). As shown in Figure 4A, differences of FC changes (RS3–RS2) in the cortico-striatal–hippocampal regions, including FC between left putamen and left motor cortical regions (M1, SMA, and SensoriMotor), and FC between left putamen and left hippocampus were observed between young and older participants. Specifically, the differences between YN and YNN in the FC changes were greater than that between ON and ONN (Table 1). To confirm the age × sleep interaction effects on the post-MSL FC changes from RS2 to RS3, and to assess the specificity of these results, a whole-brain FC approach (i.e., seed-to-voxel) was also employed to investigate FC between the pre-defined ROIs and all other voxels in the brain. As shown in Figure 4B, consistent with the ROI-to-ROI findings, we found significant age × sleep interaction effects on the putamen-seed FC changes (RS3–RS2) with motor cortical regions. Specifically, the YN group showed enhanced post-MSL FC between bilateral putamen and motor cortex regions following the nap, including the SMA (−6, −14, 44; *t* = 4.74; p_*FDR*_ = 0.001) and left M1 (−64, 4, 16; *t* = 4.92; p_*FDR*_ = 0.016) compared to the YNN group, which was significantly greater than the nap effect on FC enhancement in the older groups (ON–ONN). Although it is not the main focus of the current study, besides the interaction effects, we did not observe significant main effect on the FC changes for age group or nap condition.

**TABLE 1:** Region of interest-to-region of interest effects of [(YN–YNN) > (ON–ONN)] in the (RS3–RS2) contrast.

**Network**	**Analysis unit**	***t*-Value**	**p-unc**	**pFDR**	**pes**
Cortico-striatal–hippocampal regions	L.Putamen – L.SensoriMotor	2.78	0.002	0.048	0.111
	L.Putamen – L.SMA	2.62	0.011	0.048	0.109
	L.Putamen – L.M1	2.55	0.014	0.048	0.100
	L.Putamen – L.Hippocampus	3.21	0.002	0.031	0.136

*Threshold is p_*FDR*_ < 0.05. pes, partial E-squared; M1, primary cortex; HPC, hippocampus; Pcu, precuneus. YN, Young Nap; YNN, Young No Nap; ON, Older Nap; ONN, Older No Nap.*

#### *Post hoc* Analyses

**FIGURE 5:**
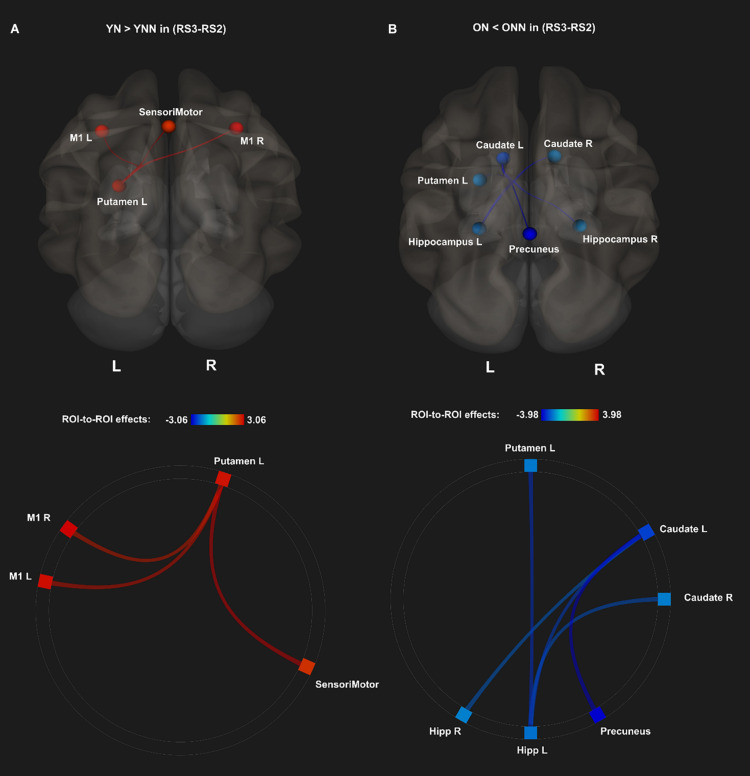
*Post hoc* analyses of ROI-to-ROI effects of sleep on MSL-related FC from post-MSL training to post-consolidation interval (RS3–RS2). **(A)** Positive FC changes (RS3–RS2) (red) for the YN as compared to the YNN, and **(B)** negative FC changes (RS3–RS2) (blue) for the ON as compared to the ONN. Thresholded at *p*_*FDR*_ < 0.05. YN, Young Nap; YNN, Young No Nap; ON, Older Nap; ONN, Older No Nap.

As shown in Figure 4 and Table 1, a daytime nap was associated with a differential contribution to post-MSL FC changes between young and older participants. Follow up analysis revealed the young group that slept showed greater FC alteration compared to the older groups regardless of sleep or wake. However, given the complexity of this interaction, it was unclear exactly where the differences originate. Therefore, we followed up this interaction by comparing the change in FC in the cortico-striatal–hippocampal regions for sleep vs. wake from RS2 to RS3 in young (YN–YNN) and older groups (ON–ONN) separately in order to unpack these significant interactions. As shown in Figure 5A, the YN group showed significantly positive FC changes (RS3–RS2) between left putamen and motor cortex regions (bilateral primary motor and sensorimotor cortex) as compared to the YNN group. In contrast, as shown in Figure 5B, as compared to the ONN participants, the ON group showed significantly negative FC changes (RS3–RS2) between the hippocampus and striatum (i.e., both the putamen and caudate), and negative FC changes between left caudate and precuneus. Detailed statistics are shown in Table 2.

**TABLE 2:** Sleep effects on post-MSL FC differences (RS3–RS2) in young and older groups.

	**FC pairs**	***t*-Value**	**p-unc**	**pFDR**	**pes**
YN–YNN	L.Putamen – SensoriMotor	3.06	0.005	0.045	0.097
	L.Putamen – L.M1	2.89	0.008	0.045	0.085
	L.Putamen – R.M1	2.80	0.010	0.045	0.106
ON–ONN	L.Hippocampus – L.Putamen	−3.32	0.003	0.022	0.111
	L.Hippocampus – L.Caudate	−3.14	0.004	0.022	0.091
	L.Hippocampus – R.Caudate	−2.86	0.008	0.029	0.075
	R.Hippocampus – L.Caudate	−2.69	0.012	0.043	0.089
	L.Caudate – Pcu	−3.98	<0.001	0.005	0.168

*Threshold is p_*FDR*_ < 0.05. pes, partial-etasquared; M1, primary cortex; HPC, hippocampus; Pcu, precuneus. YN, Young Nap; YNN, Young No Nap; ON, Older Nap; ONN, Older No Nap.*

**FIGURE 6:**
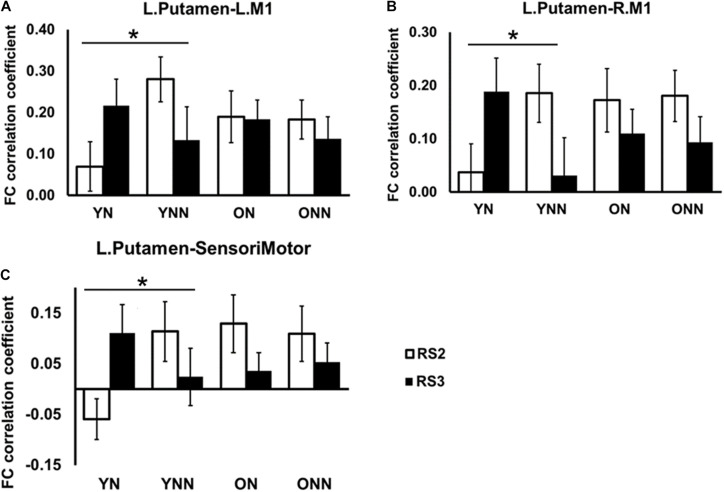
*Post hoc* analysis of the increased FC between left putamen and motor cortex in YN group following nap. Significant sleep × session interaction effects on FC were observed in the young group between **(A)** left putamen and left M1; **(B)** left putamen and right M1; **(C)** left putamen and sensorimotor, while no such effect was found in the older group. *Indicates significant sleep × session interaction effects in the young group (*pFDR < 0.05). L., left; R., right; M1, primary motor cortex. YN, Young Nap; YNN, Young No Nap; ON, Older Nap; ONN, Older No Nap.

Figures 6A–C shows the FC values between the left putamen and motor cortex areas for each group and each scan session. Significant sleep condition × session interaction effects were observed for FC between the left putamen and primary motor cortices, only in the young group, while no such effect interactions were observed in the older group. Only the YN group showed trends of increased FC in RS3 vs. RS2 between the left putamen and motor cortex areas following the nap whereas the opposite trend was observed in the no nap condition.

**FIGURE 7:**
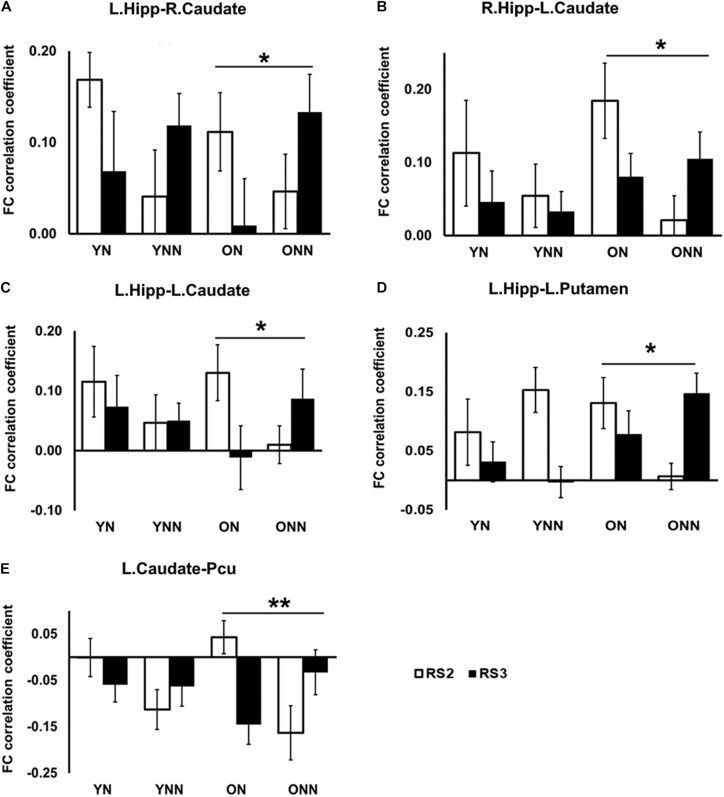
*Post hoc* analysis of the decreased FC between hippocampus and striatal area, and decreased FC between caudate and precuneus in the ON group following nap. Significant sleep × scan interaction effects on FC were observed in the older group between **(A)** left hippocampus and right caudate; **(B)** right hippocampus and left caudate; **(C)** left hippocampus and left caudate; **(D)** left hippocampus and left putamen; **(E)** left caudate and precuneus, while no such effect was found in the young group. *Indicates significant nap × session interaction effects in the older group (*pFDR < 0.05; **pFDR < 0.01). L., left; R., right; Hipp., hippocampus; Pcu, Precuneus. YN, Young Nap; YNN, Young No Nap; ON, Older Nap; ONN, Older No Nap.

Figures 7A–D shows the FC values between the hippocampus (left and right) and the striatum (caudate and putamen) for each group and each scan session. Significant sleep condition × session interaction effects were observed for FC between the hippocampus and the striatum only in the older group. In older participants, the ON group showed decreased FC in RS3 as compared to RS2 between the hippocampus and the striatum whereas the opposite tendency was observed in the ONN group. A significant sleep condition × session interaction for FC between the caudate and precuneus was also observed in the older group, while no such effect was observed in the young group (Figure 7E). The ON group showed decreased FC in RS3 compared to RS2 between left caudate, while the ONN group showed the opposite trend.

Taken together, these findings revealed the different contributions of nap to the post-MSL FC alteration. Specifically, young but not older adults displayed increased FC between putamen and motor cortex regions following a period of nap, while older, but not young, adults displayed decreased FC between hippocampus and striatal areas as well as decreased FC between the caudate and precuneus.

### Associations Between Post-Learning Motor Sequence Learning-Related Functional Connectivity Changes (RS3–RS2) and Performance Gains

**FIGURE 8:**
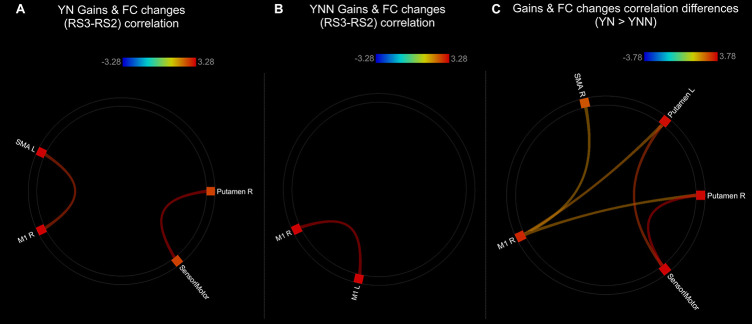
Associations between post-MSL FC changes and performance gains in **(A)** YN group; **(B)** YNN group; **(C)** YN showed stronger brain-behavioral associations than YNN group. Thresholded at *p*_*FDR*_ < 0.05. YN, Young Nap; YNN, Young No Nap.

As shown in the Figure 8, there were significant positive correlations between performance gains and FC changes (RS3–RS2) among regions within the motor network. Specifically, in YN group, FC changes between left SMA and right M1, and FC changes between right putamen and sensorimotor areas were positively correlated to performance gains (Figure 8A); while in YNN group, a correlation was found between bilateral M1 FC changes and performance gains (Figure 8B). Furthermore, we investigated group differences in the association between YN and YNN group, and the results showed that the association between FC changes within the motor network and performance gains were stronger in YN group as compared to the YNN group (Figure 8C). These results are consistent with our findings that the YN group showed greater performance gains, as well as increased FC within the motor network after a nap compared to the YNN group (Tables 3, 4).

**TABLE 3:** Significant associations between performance gains and FC changes (RS3–RS2) in young and older groups.

	**FC pairs**	***t*-Value**	**Correlation coefficient**	**p-unc**	**pFDR**
YN	R.Putamen – SensoriMotor	3.28	0.352	0.003	0.020
	R.M1 – L.SMA	2.82	0.326	0.008	0.047
YNN	R.M1 – L.M1	2.90	0.579	0.006	0.035
ON	L.Hippocampus – L.Caudate	2.50	0.456	0.013	0.042
	L.Hippocampus – R.Caudate	2.37	0.435	0.016	0.042
	L.Hippocampus – R.Putamen	2.24	0.453	0.021	0.042

*Threshold is pFDR < 0.05 (one-tailed). M1, primary cortex; SMA, supplementary motor area. YN, Young Nap; YNN, Young No Nap; ON, Older Nap; ONN, Older No Nap.*

**TABLE 4:** Differences in associations between performance gains and FC changes (RS3–RS2) between YN and YNN groups.

**FC pairs**	**Correlation coefficients**		***t*-Value**	**p-unc**	**pFDR**
	**YN**	**YNN**			
R.Putamen – SensoriMotor	0.352	−0.433	3.78	<0.001	0.003
R.Putamen – R.M1	0.125	−0.226	2.22	0.018	0.042
L.Putamen – SensoriMotor	0.054	−0.445	3.09	0.002	0.007
L.Putamen – R.M1	0.017	−0.244	2.29	0.015	0.045
R.M1 – R.SMA	0.128	−0.167	2.14	0.02	0.042

*Threshold is pFDR < 0.05 (one-tailed). M1, primary cortex; SMA, supplementary motor area. YN, Young Nap; YNN, Young No Nap; ON, Older Nap; ONN, Older No Nap.*

**FIGURE 9:**
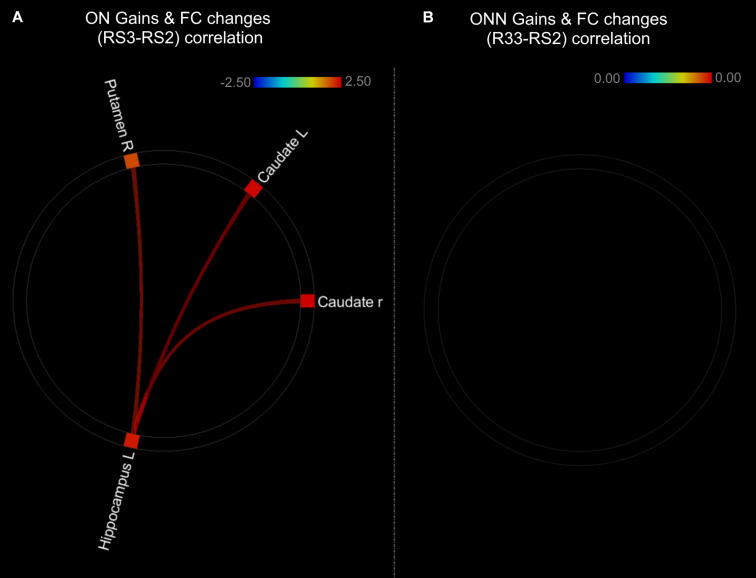
Associations between post-MSL FC changes and performance gains in **(A)** ON group; **(B)** ONN group. Thresholded at *p*_*FDR*_ < 0.05. ON, Older Nap; ONN, Older No Nap.

As shown in the Figure 9, performance gains were positively associated with FC changes (RS3–RS2) between left hippocampus and striatal regions in ON group (Figure 9A and Table 3). This result is consistent with our finding that the ON group showed decreased performance gains, as well as decreased FC between the hippocampus and striatal regions. No correlation was found between performance gains and FC changes in the ONN group (Figure 9B). However, no significant difference in the associations was found between ON and ONN groups.

## Discussion

In this study, we investigated whether sleep-dependent resting state FC during offline consolidation of MSL differed between young and older adults. After a nap, FC was increased within cortico-striatal regions in younger participants as compared to younger participants who did not nap, as well as older participant regardless of nap condition. Furthermore, MSL performance gains were correlated with FC increases in young adults, and this association was stronger in young adults who napped compared to those who did not nap. Older adults who napped showed decreased FC in hippocampal, caudate, and precuneus regions, compared to older adults who did not nap, and FC was correlated with performance in older adults who napped, but no correlations were observed in older adults who did not nap. Overall, our results suggest that younger adults exhibit a completely different pattern of FC changes during MSL consolidation over a period of rest, with sleep having further differential effects on these FC changes between age groups and is associated with behavioral gains in young participants only.

In young participants, a nap was shown to increase FC change between the putamen and motor cortical areas. Putamen-SMA connectivity is involved in motor-movement processing (Taniwaki et al., 2003) and FC between putamen and motor areas is increased during task execution (Marchand et al., 2008). Furthermore, Debas et al. (2014) found that in healthy young adults, off-line consolidation of MSL was associated with greater FC within the motor cortico-striatal network after sleep, but not an equivalent daytime period. Thus, these results support our findings in young adults, where sleep enhanced connectivity in these task-relevant areas. Failure to strengthen FC in task-related networks in older adults may reflect an overall decrease in segregated network communication, a hallmark of aging (King et al., 2018). Network segregation has been shown to decrease with age (Chong et al., 2019), possibly due to loss of white matter integrity, and gray matter volume (Marstaller et al., 2015), though not all age-related FC degradation can be explained by structural changes (Langner et al., 2015). This notion is consistent with previous work by our group showing that both age-related reductions in gray matter volume and white matter integrity are associated with reduced sleep-dependent, offline gains in MSL performance (Vien et al., 2016, 2019; Fogel et al., 2017). Interestingly, when examining changes in RS3 vs. RS2 (Figures 6, 7), young, but not older, adults displayed increased FC between putamen and motor cortex regions following a period of nap, while older, but not young, adults displayed decreased FC between hippocampus and striatal area as well as decreased FC between caudate and precuneus. This is consistent with the observed behavioral findings whereby young individuals who napped showed the greatest off-line gains, and these gains correlated with FC increases in task-relevant areas, suggesting that sleep provides a benefit at the behavioral level in youth, and the underlying brain activity supporting this benefit may be represented by changes in FC. Overall, our results suggest that sleep may primarily benefit younger individuals who show strengthened network connectivity in areas which support MSL, as has been previously observed (Jacobacci et al., 2020), and that this benefit is lost with the weakened network communication associated with normal aging.

Surprisingly, in older adults, FC change was decreased after a nap compared to wake, and this decrease was observed between the hippocampus, putamen, and caudate, as well as between the caudate and precuneus (Figure 5). Interestingly, these FC changes were positively correlated with performance gains only in older individuals who napped. Decreased communication between striatal and default mode network (DMN) areas (e.g., precuneus) after sleep in older adults may represent a shift toward network segregation in response to motor learning. Sleep-enhanced increases in striatal activity during MSL retest have previously been shown (Debas et al., 2010; Cousins et al., 2016), and a strengthening of resting state FC associated with sleep-dependent MSL consolidation has been shown to shift toward striatal networks during sleep in young adults (Vahdat et al., 2017). Age-related declines in motor performance are associated with decreased network flexibility as well as desegregation of functional networks (King et al., 2018; Monteiro et al., 2019). The degradation of hippocampal FC in older adults may result from age-related desynchronization of slow-oscillation-spindle interactions, wherein a lack of coordination between spindles and slow-oscillations may result in the failed transfer of hippocampal information during sleep (Helfrich et al., 2018).

Thus, it is possible that differences in post-nap FC-changes in our two groups reflect distinct “strategies” to consolidate a newly acquired motor skill and/or a deterioration of the optimal consolidation process with age. Our previous work has shown that sleep-dependent increases in striatal activation in older individuals is reduced as compared to young adults (Fogel et al., 2014). Moreover, sufficient recruitment of the putamen in older adults has been shown to forecast whether sleep-facilitated consolidation would occur (King et al., 2017b). Although conjectural, it is possible that decreased volume in frontal regions and the striatum (Korman et al., 2003; Raz et al., 2005) as well as disruptions in the dopaminergic system (Kaasinen and Rinne, 2002; Bäckman et al., 2010) and degradations in white matter tracts connecting the putamen to the frontal cortex (Bennett et al., 2011) and to motor cortical regions (Vien et al., 2016, 2019) might prevent reliance on the striatal aspect of the cortico-striatal–hippocampal communication, which normally supports memory consolidation in young adults. Importantly, in our present study, older adults who napped showed lower performance after sleep, whereas older adults who remained awake exhibited performance gains. This may suggest that older adults might rely on alternative, and ultimately ineffective compensatory means of neural communication during consolidation, which may involve decreases in correlated activity between the hippocampus and striatum. One intriguing possible explanation for the diminished and ultimately ineffective change in hippocampus and other brain areas involved in MSL consolidation, is that in older adults, the enhanced consolidation process is initiated during a daytime nap, but not completed, thus rending the memory trace in a vulnerable, labile state. However, this compelling possibility is somewhat speculative, and would require further direct experimental verification.

Finally, as compared to baseline, functional communication between the precuneus and the right parietal cortex was decreased following initial MSL (i.e., prior to the sleep/wake retention interval; see Figure 3 in young participants only. The precuneus and parietal cortex are both nodes of the DMN, which characteristically show greater FC at rest when internally focused (e.g., mind-wandering), in contrast to an externally focused resting state, such as being engaged in a task (Mason et al., 2007). The precuneus and parietal cortex are also both association areas involved in MSL (Gonzalez and Burke, 2018). Thus, decreased FC in this network may reflect greater MSL task-related perturbation of the DMN as a result of training in the young group, which was not observed in the older group. This finding is consistent with previous studies which demonstrated a greater decrease of activation in the DMN during a working-memory task in younger subjects compared to older subjects (Lustig et al., 2003; Pyka et al., 2009). Reduced network flexibility has been previously observed in older adults, as well as increased cortical FC and decreased striatal FC at rest (Monteiro et al., 2019). Short term motor training has been shown to increase motor network FC in young adults while decreasing FC in older adults (Mary et al., 2017; Solesio-Jofre et al., 2018). Our data supports the notion that the ability to recruit segregated task-relevant networks following training seems to be degraded with age.

Sleep is an ideal mechanism through which the brain can both strengthen and downscale neural communication. By gating external sensory information, sleep creates an opportunity for the brain to re-establish a balance of neural communication dynamics, which optimizes learning and memory (Diekelmann and Born, 2010; Chauvette et al., 2012; Grosmark et al., 2012; Rasch and Born, 2013; Aton et al., 2014; Kuhn et al., 2016). In the present study, we show evidence that changes in FC dynamics in response to MSL present differently in young compared to older individuals, and that sleep may influence the magnitude and/or direction of FC change in a brain region-specific manner. Importantly, interpretation of our current results do not take into account some potentially confounding group differences, such as a possible differences in endurance and/or sustained attention during the MSL task, as well as differences in nap length, where young adults showed longer total nap time, possibly allowing for more effective sleep-enhanced consolidation. Furthermore, the correlational nature of our analyses do not allow us to make inference about causation. In general, our results suggest that sleep preferentially strengthens FC between task-related brain areas in young participants, whereas sleep preferentially reduces connectivity between task-relevant and task-irrelevant areas in older individuals. Taken together, sleep serves to preferentially optimize communication within the striato–cortico-hippocampal in young adults, and this benefit is lost in old age.

## Data Availability Statement

The datasets presented in this article are not readily available because data is available by request to the corresponding author, following required ethical approval and inter-institutional data sharing agreements. Requests to access the datasets should be directed to SF.

## Ethics Statement

The studies involving human participants were reviewed and approved by the University of Montreal Research Ethics Board. The patients/participants provided their written informed consent to participate in this study.

## Author Contributions

ZF: analyzed the data, interpretation of results, and writing of the final manuscript. DS: made suggestions on data analysis, interpretation of results, and writing of the final manuscript. GA, BK, JC, and JD: interpretation of results and editing of manuscript. CV and HB: experimental design. SF: collected data, experimental design, analysis, interpretation of results, and editing of manuscript. All authors contributed to the article and approved the submitted version.

## Conflict of Interest

The authors declare that the research was conducted in the absence of any commercial or financial relationships that could be construed as a potential conflict of interest.

## Publisher’s Note

All claims expressed in this article are solely those of the authors and do not necessarily represent those of their affiliated organizations, or those of the publisher, the editors and the reviewers. Any product that may be evaluated in this article, or claim that may be made by its manufacturer, is not guaranteed or endorsed by the publisher.

## Footnotes

1
www.nitrc.org/projects/conn
