# Supplementary material for: Differential Effects of a Nap on Motor Sequence Learning-Related Functional Connectivity Between Young and Older Adults
Source: Front Aging Neurosci. 2021 Oct 28;13:747358. doi: 10.3389/fnagi.2021.747358 (PMC8582327; doi:10.3389/fnagi.2021.747358)
Supplement: Supplementary file 1 [file Table_1.DOCX]

| **Table S1.** Regions of interest (ROI) in cortico-striatal-hippocampal brain regions involved in MSL**.** Coordinates from CONN Toolbox (Harvard-Oxford atlas). | | | |
| --- | --- | --- | --- |
|  | X | Y | Z |
| Left Posterior parietal cortex (PPC) | -46 | -58 | 49 |
| Right (PPC) | 52 | -52 | 45 |
| Left Supplementary motor area (SMA) | -5 | -3 | 56 |
| Right SMA | 6 | -3 | 57 |
| Left Primary motor cortex (M1) | -34 | -12 | 49 |
| Right M1 | 35 | -11 | 50 |
| Sensorimotor cortex | 0 | -31 | 67 |
| Left Caudate | -13 | 9 | 10 |
| Right Caudate | 13 | 10 | 10 |
| Left Putamen | -25 | 0 | 0 |
| Right Putamen | 25 | 2 | 0 |
| Medial frontal gyrus (MFG) | -38 | 18 | 42 |
| Precuneus | 0 | 43 | -18 |
| Left Hippocampus | -25 | -23 | -14 |
| Right Hippocampus | 26 | -21 | -14 |
